# Association between serum uric acid levels and cardiovascular disease in middle-aged and elderly Chinese individuals

**DOI:** 10.1186/1471-2261-14-26

**Published:** 2014-02-25

**Authors:** Li Qin, Zhen Yang, Hongxia Gu, Shuai Lu, Qun Shi, Yin Xing, Xiaoyong Li, Rui Li, Guang Ning, Qing Su

**Affiliations:** 1 Department of Endocrinology, Xinhua hospital, School of medicine, Shanghai Jiaotong University, Kongjiang Road, Shanghai 1665, China; 2 Department of Endocrinology, Xinhua hospital Chongming branch, School of medicine, Shanghai Jiaotong University, Shanghai, China; 3 Shanghai Municipal Center For Disease Control And Prevention, Shanghai, China; 4 Department of Endocrinology, Ruijin hospital, School of medicine, Shanghai Jiaotong University, Shanghai, China

**Keywords:** Uric acid, Cardiovascular disease, Metabolic syndrome, Stroke, Coronary heart disease

## Abstract

**Background:**

A link between uric acid (UA) levels and cardiovascular diseases has been previously reported. However, its importance as a risk factor is still controversial. This study sought to determine whether elevated serum uric acid levels are associated with cardiovascular disease (CVD) in middle-aged and elderly Chinese individuals.

**Methods:**

We conducted a population-based cross-sectional study in Shanghai, with a total of 8510 participants aged ≥40 years. The CVD included diagnosed coronary heart disease (CHD) and stroke. MetS was defined according to the updated National Cholesterol Education Program Adult Treatment Panel III criteria for Asian Americans.

**Results:**

Uric acid levels were positively associated with BMI, waist circumference, triglycerides, systolic blood pressure, diastolic blood pressure, glycohemoglobin, fasting plasma glucose, postprandial 2-hour plasma glucose (all P < 0.05), and negatively associated with HDL-cholesterol (P < 0.001). The prevalence of CVD significantly increased with increasing quartiles of UA in those without MetS group (p trend < 0.001), but not necessarily increased in those with MetS. After adjustment for metabolic syndrome and other cardiovascular risk factors, multivariate logistic regression analysis showed that odds ratios (OR) for CHD, stroke, and CVD in those in the fourth quartiles were 2.34 (95% confidence interval [CI] 1.73 to 3.45), 2.18 (95% CI 1.86 to 3.28), and 2.16 (95% CI 1.80 to 3.29), respectively, compared with those in the first quartile of UA.

**Conclusions:**

Elevated serum uric acid level was associated with CVD, independent of conventional cardiovascular disease risk factors and metabolic syndrome.

## Background

During the past two decades, China has experienced rapid economic growth and the ageing of its population. Resulting changes in lifestyle and longer life expectancy have led to an increased burden of cardiovascular and other chronic diseases [1-3]. The metabolic syndrome (MetS) is characterized by a clustering of cardiovascular risk factors. Previous studies have demonstrated an association of metabolic syndrome with the development of cardiovascular disease (CVD) [3-5] and increased risk of mortality from CVD [6,7].

Uric acid is the metabolic end product of purine metabolism in humans, excess accumulation can lead to various diseases [8]. Recently, a series of controversial and conflicting findings from epidemiological studies were reported [9-21]. Previous studies have demonstrated a strong relationship between serum uric acid levels and coronary heart disease (CHD) and some studies suggested that uric acid may be an independent risk factor for cardiovascular disease [9-17]. Moreover, recently a meta-analysis showed that hyperuricemia may increase the risk of CHD events, independently of traditional CHD risk factors [18]. However, the nature of the relationship between uric acid and cardiovascular disease remains a subject of debate [19-21].

Furthermore, although previous studies have analyzed the relationship between uric acid and CVD, thus far, evidence from large sample populations about the relationship between uric acid and CVD in Chinese people is scarce. In the present study, we undertook in large-scale Chinese populations. We first investigated the association between uric acid levels and confounding factors including metabolic syndrome. In addition, we also assessed whether there is an independent association of uric acid with cardiovascular disease in individuals subdivided according to metabolic syndrome status.

## Methods

### Study population and design

**Figure 1:**
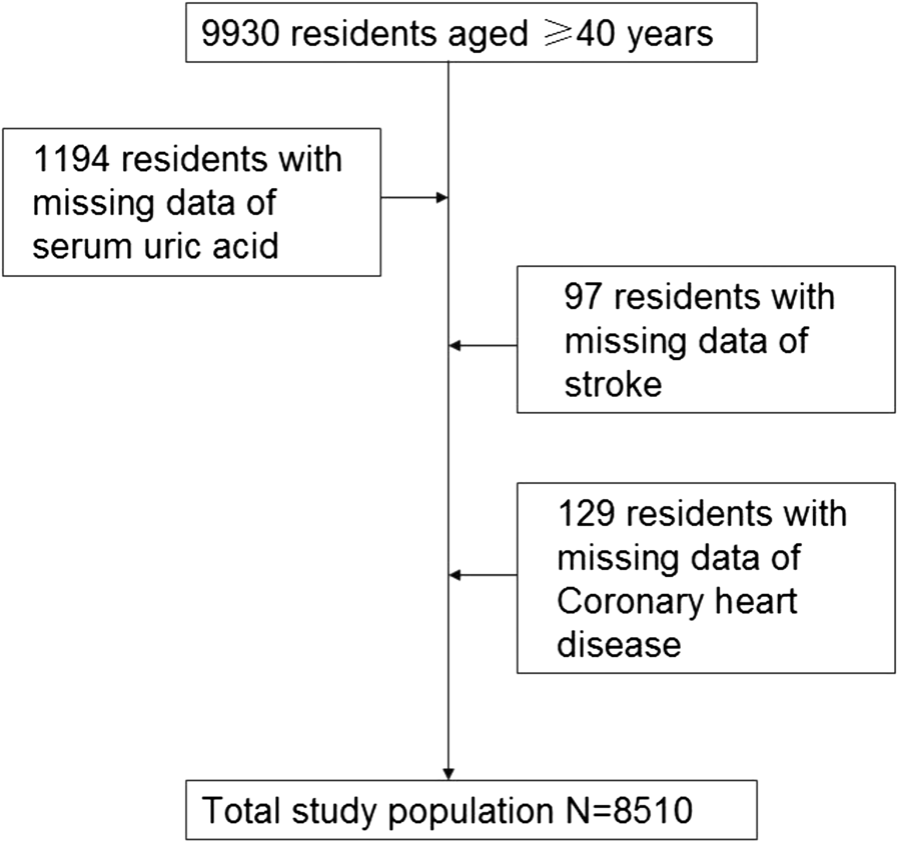
Flow chart of the recruitment and selection procedure of the study population.

From May 2011 and November 2011, a population-based cross-sectional survey (Chongming Health Investigation) was conducted in Chongming District, Shanghai, China. A two-stage stratified sampling method was used. First, 12 residential communities or streets were randomly selected from the Chongming District. Of these, 8 urban communities and 4 rural communities were chosen to represent people with high to low socioeconomic status. Secondly, Within each community/street, all eligible individuals were sampled, with the exception that in households with more than one eligible individual, one individual was randomly selected. During the recruiting phase, inhabitants aged ≥40 years in these 12 communities were invited by telephone or door-to-door visit to participate in this study. A total of 9,930 subjects completed the survey, yielding a response rate of 92.4%. Each participant signed an informed consent form before completing the questionnaire. The protocol was approved by the Institutional Review Board of Xinhua Hospital affiliated with Shanghai Jiao-Tong University School of Medicine.After excluding subjects with missing data regarding serum uric acid (n = 1194) or coronary heart disease (n = 129) or stroke (n = 97), 8510 participants were included in the final analysis (Figure 1).

### Data collection

A standardized questionnaire was used by trained physicians to collect information such as age, sex, medications, education level (6, 7–9, or ≥10 years in school). The history of chronic diseases and current use of medications were recorded. The smoking or alcohol consumption habit was defined as never, current (smoking or consuming alcohol regularly in the past 6 months), or ever (cessation of smoking or alcohol consumption for more than 6 months). The type, amount, and frequency of alcohol consumption were collected. Based on the alcohol content of the beverages reported, mean daily alcohol consumption was calculated and expressed in grams per day. Physical activity at leisure time was estimated using the short form of the International Physical Activity Questionnaire by adding questions on frequency and duration of moderate and vigorous activities and walking [22].

Anthropometric measurements were performed by the trained personnel using a standardized protocol. Height was measured to the nearest 0.1 cm, and weight was recorded to the nearest 0.1 kg while participants were wearing lightweight clothing and no shoes. Body mass index (BMI) was defined as body weight in kilograms divided by height squared in meters. Waist circumference (WC) was measured to the nearest 0.1 cm at the umbilical level with the participant in a standing position. Hip circumference was measured over the widest part of the gluteal region, and the waist-to-hip ratio was calculated as a measure of central obesity. Three blood pressure recordings were obtained from the right arm of patients in a sitting position after 30 min of rest; measurements were taken in 5-min intervals, and mean values were calculated.

### Biochemical measurements

Peripheral venous blood samples were collected after an overnight fast. The fasting glucose, glucose 2 h after oral glucose tolerance test were measured with the use of the glucose oxidase method on an autoanalyzer (Modular P800; Roche, Basel, Switzerland). Triglycerides (TG), total cholesterol (TC), high-density lipoprotein cholesterol (HDL-c), low-density lipoprotein cholesterol (LDL-c), and serum uric acid were measured using chemiluminescence methods on the autoanalyzer (Modular E170; Roche). Glycated hemoglobin(HbA1c) was measured with the use of the Chromatography method on an autoanalyzer (D10; Bio-Rad, USA).

### Diagnosis of CVD

Hypertension was defined as diastolic blood pressure ≥90 mm Hg, systolic blood pressure of ≥140 mm Hg, or current medication for hypertension (as defined by WHO 1999) [16]. Coronary heart disease (CHD) and stroke were defined using the WHO MONICA criteria [23]. Myocardial infarction was diagnosed by a representative set of electrocardiogram, cardiac enzyme values, and typical symptoms. Angina was defined as use of nitroglycerine, experience of typical chest pain, and electrocardiogram changes compatible with ischemic heart disease. Strokes were defined as events requiring hospitalization; this information was verified from local hospital records, and 82% of the cases were confirmed using computed tomography and magnetic resonance imaging. Cardiovascular disease in the present study was defined by the presence of one or more of these two outcomes: CHD and stroke. Subjects with a fasting plasma glucose level of 7.0 mmol/l and/or a 2-h plasma glucose level of 11.1 mmol/l during an oral glucose tolerance test and/or who were receiving antidiabetic medications were diagnosed with diabetes mellitus. The diagnosis of cardiovascular events was based on self-reports, confirmed by hospital medical records and further clinical examinations carried out at the time of the survey (including electrocardiogram and ankle-arm systolic blood pressure index).

### Definition of MetS

The MetS was defined based upon the updated NCEP-ATPIII for Asian Americans [24] as presenting 3 or more of the following components: 1) waist cirumference ≥90 cm for men or ≥80 cm for women; 2) triglycerides ≥1.7 mmol/l; 3) HDL cholesterol <1.03 mmol/l for men or <1.30 mmol/l for women; 4) blood pressure ≥130/85 mm Hg or current use of anti-hypertensive medications; and 5) fasting glucose ≥5.6 mmol/l or previously diagnosed type 2 diabetes or on oral antidiabetic agents or insulin.

### Statistical analysis

Normally distributed data were expressed as means ± SD, whereas variables with a skewed distribution were reported as median (interquartile range) and log transformed to approximate normality before analysis. Categorical variables were represented by frequency and percentage. The association of demographic, medical, metabolic and clinical characteristics with serum uric acid quartiles was assessed using multivariate linear or logistic regression analyses, adjusting for age and gender, for continuous and dichotomous variables, respectively. The correlations among serum uric acid, anthropometric indices and metabolic features adjusting for age and gender were obtained using Spearman partial correlation analysis. Sampling weights was used when estimating prevalence of CVD. The trend for age- and sex-adjusted prevalence of CVD according to quartile of uric acid and the presence of metabolic syndrome were analysed using linear regression model. Multivariate logistic regression models were used to estimate the odds ratios (ORs) for CHD, stroke and CVD. Potential confounding variables including age, gender, smoking, alcohol drinking, physical activity, educational level, education, BMI, and metabolic syndrome were controlled in the regression models. All statistical analysis were performed with the SPSS Statistical Package (version 15.0; SPSS Inc., Chicago, IL). P < 0.05 was considered statistically significant.

## Results

### Characteristics of participants according to serum uric acid quartiles

Included were 8510 individuals who had both uric acid and CVD assessments (Figure 1). The mean age of this cohort was 55.90 ± 7.89 years, and approximately 32% were men. Baseline demographic and medical characteristics for both genders combined and divided by uric acid quartiles are presented in Table 1. Higher uric acid levels were associated with higher proportion of metabolic syndrome, proportion of men, age, smoking, alcohol consumption, systolic blood pressure, diastolic blood pressure, BMI, waist circumference, hip circumference, waist to hip ratio, fasting plasma glucose, postprandial 2-hour plasma glucose, glycohemoglobin, triglycerides, total cholesterol, LDL-cholesterol, presence of stroke, presence of CHD, hypertension, diabetes (all P < 0.05). In contrast, the patients with higher uric acid levels displayed lower levels of HDL cholesterol (P < 0.001) (Table 1).

**Table 1:** Characteristics of study participants according to uric acid quartiles

**Characteristics**	**Q1**	**Q2**	**Q3**	**Q4**	**P value**
	**(n = 2428)**	**(n = 2114)**	**(n = 1986)**	**(n = 1982)**	
	**≤0.2**	**0.21-0.24**	**0.25-0.29**	**≥0.30**	
MetS (%)	927 (38.2)	1112 (52.6)	1210 (60.9)	1410 (71.1)	<0.001
Uric acid (mmol/L)	0.18 (0.16-0.19)	0.22 (0.22-0.24)	0.27 (0.26-0.28)	0.34 (0.31-0.37)	<0.001
Male n (%)	316 (13.02)	500 (23.65)	705 (35.52)	1205 (60.80)	<0.001
Age (yrs)^b^	53.29 ± 7.97	55.72 ± 7.75	57.25 ± 7.45	57.93 ± 7.46	<0.001
Smoking (yes)	413 (17.02)	411 (19.44)	501 (25.23)	680 (34.31)	<0.001
Alcohol (yes)	524 (21.59)	514 (24.31)	579 (29.15)	852 (42.99)	<0.001
SBP (mmHg)	125 ± 20	129 ± 21	132 ± 20	135 ± 19	<0.001
DBP (mmHg)	78 ± 10	80 ± 10	81 ± 11	83 ± 10	<0.001
BMI (kg/m^2^)	23.41 ± 3.19	24.76 ± 8.45	25.53 ± 11.29	25.79 ± 4.49	<0.001
Waist circumference (cm)	80 ± 10	84 ± 12	86 ± 10	89 ± 9	<0.001
Hip circumference (cm)	94.07 ± 7.40	95.85 ± 7.00	96.85 ± 6.78	98.09 ± 6.89	<0.001
Fasting plasma glucose (mmol/L)	6.05 ± 1.50	6.39 ± 1.89	6.50 ± 1.80	6.53 ± 1.61	<0.001
P2hPG (mmol/L)	7.95 ± 3.49	8.88 ± 4.16	9.26 ± 4.04	9.36 ± 3.91	<0.001
HbA1C	5.84 ± 0.97	6.01 ± 1.10	6.05 ± 1.07	6.02 ± 0.94	0.021
Triglycerides (mmol/L)^c^	1.09 (0.82-1.52)	1.36 (0.98-1.93)	1.49 (1.06-2.24)	1.86 (1.29-2.67)	<0.001
Total cholesterol (mmol/L)	4.59 ± 0.99	4.79 ± 0.99	4.81 ± 1.01	4.88 ± 1.05	<0.001
LDL cholesterol (mmol/L)	2.56 ± 0.75	2.66 ± 0.76	2.72 ± 0.79	2.72 ± 0.78	<0.001
HDL cholesterol (mmol/L)	1.33 ± 0.33	1.27 ± 0.31	1.22 ± 0.31	1.17 ± 0.30	<0.001
Physical activity					0.191
Low	1795 (73.96)	1538 (72.75)	1453 (73.16)	1395 (70.38)	
Moderate	487 (20.07)	435 (20.58)	397 (19.99)	438 (22.10)	
High	145 (5.97)	141 (6.67)	136 (6.85)	149 (7.52)	
Education					0.061
0-6	520 (21.43)	516 (24.41)	497 (25.03)	464 (23.41)	
7-9	1217 (50.14)	1048 (49.57)	989 (47.80)	988 (49.85)	
≥10	690 (28.43)	550 (26.02)	500 (27.17)	530 (26.74)	
Stroke	69 (2.84)	84 (3.97)	99 (4.98)	168 (8.48)	<0.001
Coronary heart disease	149 (6.14)	171 (8.09)	233 (11.73)	379 (19.12)	<0.001
Hypertension	1029 (42.40)	1109 (52.46)	1213 (61.08)	1386 (69.93)	<0.001
Diabetes mellitus	319 (13.14)	453 (21.43)	574 (28.90)	622 (31.38)	<0.001
Anti-hypertension medications	342 (14.09)	351 (16.60)	394 (19.84)	448 (22.60)	<0.001
Anti-diabetic medications	110 (4.53)	111 (5.25)	140 (7.05)	119 (6.00)	0.003
Lipid-lowering medications	65 (2.68)	71 (3.36)	62 (3.12)	57 (2.88)	0.571

SBP, systolic blood pressure; DBP, diastolic blood pressure; BMI, body mass index; P2hPG, postprandial 2-hour plasma glucose; HbA1C, Glycated hemoglobin; LDL, Low-density lipoprotein; HDL, high--density lipoprotein.

^a^Data are presented as mean ± SD, median (interquartile range), or number (percent); P value was calculated after adjustment for age, gender.

^b^Not adjusted for itself.

^c^This variables was log transformed before analysis.

### Association between serum uric acid and CHD, stroke and CVD

**Figure 2:**
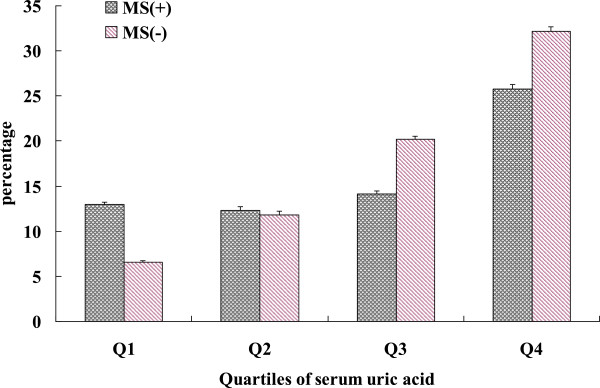
**Prevalence of CVD according to quartiles of uric acid and the presence of metabolic syndrome (adjusted for age, gender, alcohol drinking, smoking, education, physical activity, TC, LDL).** Data are expressed as percentages ± SE. P < 0.001 for trend in MS(−) group, and p = 0.217 for trend in MS(+) group.

Partial Spearman correlation analysis demonstrated the strongest correlation between uric acid and triglycerides among various metabolic features (Table 2). Moreover there was strong partial correlation between uric acid and BMI, TC, Waist circumference (WC), systolic blood pressure (SBP) and diastolic blood pressure (DBP), adjusting for age and gender, (all P < 0.001) (Table 2). As presented in Table 3, the ORs for CHD, stroke and CVD were higher with increasing uric acid quartiles (all p < 0.001 for linear trend). In the highest uric acid quartile, the adjusted ORs of CHD, stroke and CVD were 2.34 (95% confidence interval [CI] 1.73 to 3.45), 2.18 (95% CI 1.86 to 3.27), and 2.16 (95% CI 1.80 to 3.29), respectively, compared with those in the first quartile of UA (Table 3).We further analyzed the prevalence of CVD according to quartile of uric acid and the presence of metabolic syndrome. After adjustments for age, gender, alcohol drinking, smoking, education, physical activity, TC, LDL, the prevalence of CVD in subjects with metabolic syndrome increased with uric acid quartile (p < 0.001 for linear trend) (Figure 2). No trend was observed in subject without metabolic syndrome (p = 0.217 for linear trend).

**Table 2:** Partial Spearman correlation coefficients among uric acid, anthropometric indicators, and metabolic features

	**Uric acid**	**BMI**	**WC**	**HC**	**WHR**	**SBP**	**DBP**	**FPG**	**P2hPG**	**HbA1c**	**TC**	**TG**	**HDL-c**
BMI	0.11^b^												
WC	0.21^b^	0.33^b^											
HC	0.11^b^	0.34^b^	0.67^b^										
WHR	0.08^b^	0.11^b^	0.47^b^	−0.11^b^									
SBP	0.10^b^	0.11^b^	0.23^b^	0.17^b^	0.12^b^								
DBP	0.14^b^	0.11^b^	0.23^b^	0.19^b^	0.09^b^	0.62^b^							
FPG	0.04^b^	0.02^c^	0.09^b^	0.03^c^	0.07^b^	0.14^b^	0.10^b^						
P2hPG	0.09^b^	0.05^b^	0.12^b^	0.07^b^	0.08^b^	0.15^b^	0.11^b^	0.76^b^					
HbA1c	0.02^c^	0.02^c^	0.07^b^	0.03^c^	0.05^b^	0.05^b^	0.03^c^	0.82^b^	0.71^b^				
TC	0.12^b^	0.02	0.08^b^	0.05^b^	0.04^b^	0.12^b^	0.13^b^	0.12^b^	0.10^b^	0.08^b^			
TG	0.27^b^	0.09^b^	0.21^b^	0.13^b^	0.11^b^	0.11^b^	0.15^b^	0.18^b^	0.21^b^	0.14^b^	0.23^b^		
HDL-c	−0.18^b^	−0.09^b^	−0.17^b^	−0.12^b^	−0.07^b^	0.02^c^	0.00	−0.04^b^	−0.09^b^	−0.10^b^	0.49^b^	−0.29^b^	
LDL-c	0.08^b^	0.02	0.06^b^	0.07^b^	0.02^c^	0.07^b^	0.08^b^	0.08^b^	0.05^b^	0.08^b^	0.86^b^	−0.04^c^	0.33^b^

BMI, body mass index; WC, Waist circumference, HC, Hip circumference; WHR, Waist-hip-ratio; SBP, systolic blood pressure; DBP, diastolic blood pressure; FPG, fasting plasma glucose; P2hPG, postprandial 2-hour plasma glucose; HbA1C, Glycated hemoglobin; TC, Triglycerides; Total cholesterol; TG, LDL, Low-density lipoprotein; HDL, high--density lipoprotein.

^a^All correlation coefficients were calculated after adjustment for age, gender.

^b^P < 0.001.

^c^P < 0.05.

**Table 3:** Odds ratios and 95% confidence interval for cardiovascular disease according to quartile of serum uric acid

	**Q1**	**Q2**	**Q3**	**Q4**	**P value for trend**
	**OR (95% CI)**	**OR (95% CI)**	**OR (95% CI)**	**OR (95% CI)**	
CHD					
Model 1^*^	1	1.73 (1.36-2.19)	1.95 (1.70-2.45)	2.56 (1.95-3.89)	<0.001
Model 2^†^	1	1.71 (1.35-2.17)	1.90 (1.63-2.42)	2.45 (1.82-3.56)	<0.001
Model 3^‡^	1	1.65 (1.28-2.14)	1.82 (1.63-2.35)	2.34 (1.73-3.45)	<0.001
Stroke					
Model 1^*^	1	1.40 (1.02-1.96)	1.74 (1.29-2.43)	2.41 (2.02-3.65)	<0.001
Model 2^†^	1	1.36 (1.03-1.93)	1.67 (1.27-2.41)	2.27 (1.92-3.39)	<0.001
Model 3^‡^	1	1.30 (1.02-1.81)	1.60 (1.23-2.29)	2.18 (1.86-3.28)	<0.001
CVD					
Model 1^*^	1	1.39 (1.14-1.68)	1.87 (1.54-2.44)	2.50 (2.09-3.47)	<0.001
Model 2^†^	1	1.31 (1.11-1.62)	1.77 (1.49-2.48)	2.40 (2.06-3.40)	<0.001
Model 3^‡^	1	1.28 (1.09-1.56)	1.64 (1.35-2.38)	2.16 (1.80-3.29)	<0.001

^*^Model 1 adjusted for age and gender.

^†^Model 2 further adjusted for alcohol drinking, smoking, education, physical activity, TC, LDL and treatments (including antihypertensive therapy, antihyperlipidemic therapy and antihyperglycemic therapy).

^‡^Model 3 further adjusted for metabolic syndrome and BMI.

## Discussion

In the present study, we found that serum uric acid levels showed association with the risk of CVD from the cross-sectional data in middle-aged and elderly Chinese individuals, independent of conventional cardiovascular risk factors including components of metabolic syndrome.

Several previous studies in literature have documented the relationship between serum uric acid levels and cardiovascular diseases [9-17,25]. Increased serum uric acid was found to be associated with important risk factors for atherosclerosis like hypertension [26-29], abdominal obesity [28], diabetes mellitus [29,30], the metabolic syndrome [17], hypertriglyceridemia [31], endothelial dysfunction [32] and renal failure [33]. However, whether uric acid is an independent risk factor for cardiovascular mortality is still a controversy. Difficulties in determining whether uric acid should be considered a cardiovascular risk factor may be explained by its frequent association with other cardiovascular risk factors [34] for which uric acid is considered as a risk marker or epiphenomenon or even an adaptive change to protect from atherosclerosis due to its antioxidant properties [35] and the controversial and conflicting findings from epidemiological studies [36].

The metabolic syndrome is characterized by a clustering of cardiovascular risk factors, including abdominal obesity, high blood pressure, increased glucose concentration, and dyslipidemia, is a common basis for the development of atherosclerosis, especially CVD [37-39]. Moreover, previous studies claimed that hyperuricemia was a new component of metabolic syndrome. Furthermore, in our study, the proportion of metabolic syndrome is in parallel with increasing serum uric acid quartiles. Thus, in order to explore whether elevated serum uric acid levels are associated with CVD independent of metabolic syndrome, we further analyzed the prevalence of CVD according to quartile of uric acid and the presence of metabolic syndrome. We found that prevalence of CVD is significantly increased with increasing quartiles of uric acid in without MetS group (p trend < 0.001), but not necessarily increased in those with metabolic syndrome. Additional factors besides metabolic syndrome may also play a key role in the development of CVD in hyperuricemia subjects, which requires further research.

The present study may have some implications. In particular, this study may strengthen the need for interventional studies with uric acid-lowering therapies to maintain UA levels in the lower safe range. Moreover, allopurinol – a xanthine oxidase inhibitor – has been shown recently to significantly improve endothelial function and abolish vascular oxidative stress [40], has a clinically relevant antiischaemic effect and has been well tolerated in patients with angina [41]. In analogy with homocysteine-lowering therapy interventional studies with UA-lowering agents, apart from exploring clinical benefits of these agents, may provide valuable additional information regarding causality in the uric acid–cardiovascular disease relationship.

The major strength of our present study was that the large sample size and representative sample with a relatively high response rate. Several limitations of our study have also to be addressed. First, due to the cross-sectional nature of the present study, no causal relationships can be established. Large prospective studies are in urgent need to confirm the relationship between serum uric acid levels and CVD. Second, although most potential confounders were carefully controlled, since some of the study subjects may had several chronic disease, we could not eliminate the possible effect of underlying diseases and medications used for these diseases on the present findings.

## Conclusion

In conclusion, the present study showed that elevated serum uric acid level was associated with CVD, independent of conventional CVD risk factors and the presence of metabolic syndrome. The study added more evidence to the notion that the risk of CVD increased in subjects with hyperuricemia.

## Competing interests

The authors declare that they have no competing interests.

## Authors’ contributions

Conceived and designed the experiments: QS. Analyzed the data: LQ, ZY. Contributed reagents/materials/analysis tools: LQ, ZY, HG, SL, QS, YX, XL, RL, GN. Wrote the paper: LQ, ZY. All authors read and approved the final manuscript.

## Pre-publication history

The pre-publication history for this paper can be accessed here:

http://www.biomedcentral.com/1471-2261/14/26/prepub

## References

[B1] Wu Z Yao C Zhao D Wu G Wang W Liu J Zeng Z Wu Y Sino-MONICA project: a collaborative study on trends and determinants in cardiovascular diseases in China, part I: morbidity and mortality monitoring Circulation 2001 103 462 468 10.1161/01.CIR.103.3.462 11157701

[B2] Yang Z Wang X Wen J Ye Z Li Q He M Lu B Ling C Wu S Hu R Prevalence of non-alcoholic fatty liver disease and its relation to hypoadiponectinaemia in the middle-aged and elderly Chinese population Arch Med Sci 2011 665 672 22291803 10.5114/aoms.2011.24137 PMC3258786

[B3] He Y Jiang B Wang J Feng K Chang Q Fan L Li X Hu FB Prevalence of the metabolic syndrome and its relation to cardiovascular disease in an elderly Chinese population J Am Coll Cardiol 2006 47 1588 1594 10.1016/j.jacc.2005.11.074 16630995

[B4] Haffner SM Valdez RA Hazuda HP Mitchell BD Morales PA Stern MP Prospective analysis of the insulin-resistance syndrome (Syndrome X) Diabetes 1992 41 715 722 10.2337/diab.41.6.715 1587398

[B5] Isomaa B Almgren P Tuomi T Forsén B Lahti K Nissén M Taskinen MR Groop L Cardiovascular morbidity and mortality associated with the metabolic syndrome Diabetes Care 2001 24 683 689 10.2337/diacare.24.4.683 11315831

[B6] Trevisan M Liu J Bahsas FB Menotti A Syndrome X and mortality:a population-based study. Risk Factor and Life Expectancy Research Group Am J Epidemiol 1998 148 958 966 10.1093/oxfordjournals.aje.a009572 9829867

[B7] Lakka HM Laaksonen DE Lakka TA Niskanen LK Kumpusalo E Tuomilehto J Salonen JT The metabolic syndrome and total and cardiovascular disease mortality in middle-aged men JAMA 2002 288 2709 2716 10.1001/jama.288.21.2709 12460094

[B8] So A Thorens B Uric acid transport and disease J Clin Invest 2010 120 1791 1799 10.1172/JCI42344 20516647 PMC2877959

[B9] Neogi T Ellison RC Hunt S Terkeltaub R Felson DT Zhang Y Serum uric acid is associated with carotid plaques: the National Heart, Lung, and Blood Institute Family Heart Study J Rheumatol 2009 36 378 384 19012359 10.3899/jrheum.080646 PMC2731484

[B10] Bos MJ Koudstaal PJ Hofman A Witteman JC Breteler MM Uric acid is a risk factor for myocardial infarction and stroke: the Rotterdam study Stroke 2006 37 1503 1507 10.1161/01.STR.0000221716.55088.d4 16675740

[B11] Franse LV Pahor M Di Bari M Shorr RI Wan JY Somes GW Applegate WB Serum uric acid, diuretic treatment and risk of cardiovascular events in the Systolic Hypertension in the Elderly Program (SHEP) J Hypertens 2000 18 1149 1154 10.1097/00004872-200018080-00021 10954008

[B12] Juraschek SP Tunstall-Pedoe H Woodward M Serum uric acid and the risk of mortality during 23 years follow-up in the Scottish Heart Health Extended Cohort Study Atherosclerosis 2014 233 623 629 10.1016/j.atherosclerosis.2014.01.026 24534458 PMC5590638

[B13] Ito H Abe M Mifune M Oshikiri K Antoku S Takeuchi Y Togane M Hyperuricemia is independently associated with coronary heart disease and renal dysfunction in patients with type 2 diabetes mellitus PLoS One 2011 6 e27817 10.1371/journal.pone.0027817 22125626 PMC3220675

[B14] Freedman DS Williamson DF Gunter EW Byers T Relation of serum uric acid to mortality and ischemic heart disease. The NHANES I Epidemiologic Follow-up Study Am J Epidemiol 1995 141 637 644 7702038 10.1093/oxfordjournals.aje.a117479

[B15] Krishnan E Baker JF Furst DE Schumacher HR Gout and the risk of acute myocardial infarction Arthritis Rheum 2006 54 2688 2696 10.1002/art.22014 16871533

[B16] Verdecchia P Schillaci G Reboldi G Santeusanio F Porcellati C Brunetti P Relation between serum uric acid and risk of cardiovascular disease in essential hypertension. The PIUMA study Hypertension 2000 36 1072 1078 10.1161/01.HYP.36.6.1072 11116127

[B17] Li Q Yang Z Lu B Wen J Ye Z Chen L He M Tao X Zhang W Huang Y Zhang Z Qu S Hu R Serum uric acid level and its association with metabolic syndrome and carotid atherosclerosis in patients with type 2 diabetes Cardiovasc Diabetol 2011 10 72 10.1186/1475-2840-10-72 21816063 PMC3163178

[B18] Kim SY Guevara JP Kim KM Choi HK Heitjan DF Albert DA Hyperuricemia and coronary heart disease: a systematic review and meta-analysis Arthritis Care Res (Hoboken) 2010 62 170 180 20191515 10.1002/acr.20065 PMC3156692

[B19] Culleton BF Larson MG Kannel WB Levy D Serum uric acid and risk for cardiovascular disease and death: the Framingham Heart Study Ann Intern Med 1999 131 7 13 10.7326/0003-4819-131-1-199907060-00003 10391820

[B20] Brand FN McGee DL Kannel WB Stokes J 3rd Castelli WP Hyperuricemia as a risk factor of coronary heart disease: The Framingham Study Am J Epidemiol 1985 121 11 18 3964986 10.1093/oxfordjournals.aje.a113972

[B21] Moriarity JT Folsom AR Iribarren C Nieto FJ Rosamond WD Serum uric acid and risk of coronary heart disease: Atherosclerosis Risk in Communities (ARIC) Study Ann Epidemiol 2000 10 136 143 10813506 10.1016/s1047-2797(99)00037-x

[B22] Guidelines for data processing and analysis of the International Physical Activity Questionnaire (IPAQ) 2006 Available at: http://www.ipaq.ki.se/25376692

[B23] WHO/MONICA-Project Multinational Monitoring of Trends andDeterminants in Cardiovascular Diseases (MONICA Project) and Manual of Operation 1983 Geneva, Switzerland: World Health Organization, Cardiovascular Disease Unit

[B24] Liu J Grundy SM Wang W Smith SC Vega GL Wu Z Zeng Z Wang W Zhao D Ethnic-Specific Criteria for the Metabolic Syndrome Diabetes Care 2006 29 1414 1416 10.2337/dc06-0481 16732037

[B25] Takayama S Kawamoto R Kusunoki T Abe M Onji M Uric acid is an independent risk factor for carotid atherosclerosis in a Japanese elderly population without metabolic syndrome Cardiovasc Diabetol 2012 11 2 10.1186/1475-2840-11-2 22234039 PMC3293733

[B26] Perlstein TS Gumieniak O Williams GH Sparrow D Vokonas PS Gaziano M Weiss ST Litonjua AA Uric acid and the development of hypertension: the normative aging study Hypertension 2006 48 1031 1036 10.1161/01.HYP.0000248752.08807.4c 17060508

[B27] Shankar A Klein R Klein BE Nieto FJ The association between serum uric acid level and long-term incidence of hypertension: Population-based cohort study J Hum Hypertens 2006 20 937 945 10.1038/sj.jhh.1002095 17024135

[B28] Masuo K Kawaguchi H Mikami H Ogihara T Tuck ML Serum uric acid and plasma norepinephrine concentrations predict subsequent weight gain and blood pressure elevation Hypertension 2003 42 474 480 10.1161/01.HYP.0000091371.53502.D3 12953019

[B29] Nakanishi N Okamoto M Yoshida H Matsuo Y Suzuki K Tatara K Serum uric acid and risk for development of hypertension and impaired fasting glucose or Type II diabetes in Japanese male office workers Eur J Epidemiol 2003 18 523 530 12908717 10.1023/a:1024600905574

[B30] Dehghan A Van Hoek M Sijbrands EJ Hofman A Witteman JC High serum uric acid as a novel risk factor for type 2 diabetes Diabetes Care 2008 31 361 362 17977935 10.2337/dc07-1276

[B31] Rathmann W Funkhouser E Dyer AR Roseman JM Relations of hyperuricemia with the various components of the insulin resistance syndrome in young black and white adults: the CARDIA study. Coronary Artery Risk Development in Young Adults Ann Epidemiol 1998 8 250 261 9590604 10.1016/s1047-2797(97)00204-4

[B32] Khosla UM Zharikov S Finch JL Nakagawa T Roncal C Mu W Krotova K Block ER Prabhakar S Johnson RJ Hyperuricemia induces endothelial dysfunction Kidney Int 2005 67 1739 1742 10.1111/j.1523-1755.2005.00273.x 15840020

[B33] Iseki K Ikemiya Y Inoue T Iseki C Kinjo K Takishita S Significance of hyperuricemia as a risk factor for developing ESRD in a screened cohort Am J Kidney Dis 2004 44 642 650 10.1053/j.ajkd.2004.06.006 15384015

[B34] Davies KJ Sevanian A Muakkassah-Kelly SF Hochstein P Uric acid-iron ion complexes. A new aspect of the antioxidant functions of uric acid Biochem J 1986 235 747 754 3753442 10.1042/bj2350747 PMC1146751

[B35] Fang J Alderman MH Serum uric acid and cardiovascular mortality the NHANES I epidemiologic follow-up study, 1971–1992. National Health and Nutrition Examination Survey JAMA 2000 283 2404 2410 10.1001/jama.283.18.2404 10815083

[B36] Niskanen LK Laaksonen DE Nyyssonen K Alfthan G Lakka HM Lakka TA Uric acid level as a risk factor for cardiovascular and all-cause mortality in middle-aged men: a prospective cohort study Arch Intern Med 2004 164 1546 1551 10.1001/archinte.164.14.1546 15277287

[B37] Chen L Yang Z Lu B Li Q Ye Z He M Huang Y Wang X Zhang Z Wen J Liu C Qu S Hu R:Serum CXC ligand 5 is a new marker of subclinical atherosclerosis in type 2 diabetes Clin Endocrinol (Oxf) 2011 75 766 770 10.1111/j.1365-2265.2011.04119.x 21609350

[B38] Yang Z Zhang Z Wen J Wang X Lu B Yang Z Zhang W Wang M Feng X Ling C Wu S Hu R Elevated serum chemokine CXC ligand 5 levels are associated with hypercholesterolemia but not a worsening of insulin resistance in Chinese people J Clin Endocrinol Metab 2010 95 3926 3932 10.1210/jc.2009-2194 20501684

[B39] Huang Y Yang Z Ye Z Li Q Wen J Tao X Chen L He M Wang X Lu B Zhang Z Zhang W Qu S Hu R Lipocalin-2, glucose metabolism and chronic low-grade systemic inflammation in Chinese people Cardiovasc Diabetol 2012 11 11 10.1186/1475-2840-11-11 22292925 PMC3295671

[B40] Rajendra NS Ireland S George J Belch JJ Lang CC Struthers AD Mechanistic insights into the therapeutic use of high-dose allopurinol in angina pectoris J Am Coll Cardiol 2011 58 820 828 10.1016/j.jacc.2010.12.052 21835317

[B41] Noman A Ang DS Ogston S Lang CC Struthers AD Effect of high-dose allopurinol on exercise in patients with chronic stable angina: a randomised, placebo controlled crossover trial Lancet 2010 375 2161 2167 10.1016/S0140-6736(10)60391-1 20542554 PMC2890860

